# Long‐term changes in bone mineral density in postoperative patients with esophageal cancer

**DOI:** 10.1002/ags3.12640

**Published:** 2022-11-29

**Authors:** Takahito Sugase, Keijiro Sugimura, Takashi Kanemura, Tomohira Takeoka, Masaaki Yamamoto, Naoki Shinno, Hisashi Hara, Takeshi Omori, Masayoshi Yasui, Hiroshi Miyata

**Affiliations:** ^1^ Department of Gastroenterological Surgery Osaka International Cancer Institute Osaka Japan; ^2^ Department of Gastroenterological Surgery Kansai Rosai Hospital Amagasaki Japan

**Keywords:** bone mineral density, esophageal cancer, esophagectomy, osteoporosis, weight loss

## Abstract

**Aim:**

The aim of this study was to investigate long‐term changes in bone mineral density (BMD) after esophagectomy, identify the risk factors for postoperative osteoporosis in patients with esophageal cancer and survival outcomes related to osteoporosis.

**Methods:**

We retrospectively evaluated BMD changes for 197 consecutive patients with thoracic esophageal cancer who were disease‐free for 5 years after radical esophagectomy. Osteoporosis was diagnosed using computed tomography with an L1 attenuation threshold of ≤110 HU. Survival analysis was performed on 381 consecutive patients with 5‐year follow‐up after radical esophagectomy.

**Results:**

BMD decreased annually after esophagectomy. The median attenuation (HU) was 134.2 before surgery and 135.2, 127.4, 123.3, 115.2, 105.6, and 102.4 at 6 months and 1, 2, 3, 4, and 5 years after surgery, respectively. Osteoporosis was diagnosed in 25.9% patients before surgery and 23.3%, 29.4%, 40.1%, 46.7%, 54.8%, and 60.4% patients with osteoporosis were observed at 6 months and 1, 2, 3, 4, and 5 years after surgery, respectively. Postoperative BMD did not decrease in patients aged ≤54 years, those who had never been smokers, and those with no weight loss after esophagectomy. Multivariate analysis identified that age (≥65 years) at surgery and smoking history were independent risk factors for osteoporosis at 5 years after esophagectomy. Patients with preoperative osteoporosis tended to have worse prognosis in disease‐free survival and overall survival than those without osteoporosis, who were more likely to die due to non‐esophageal cancer.

**Conclusion:**

Esophageal cancer survivors are more likely to develop osteoporosis after esophagectomy, and preoperative osteoporosis might be associated with prognosis.

## Graphical abstract

The aim of this study was to evaluate long‐term changes in bone mineral density after esophagectomy and identify the risk factors for postoperative osteoporosis. Esophageal cancer survivors are more likely to develop osteoporosis after esophagectomy, with risk factors including both preoperative patient factors and postoperative factors.

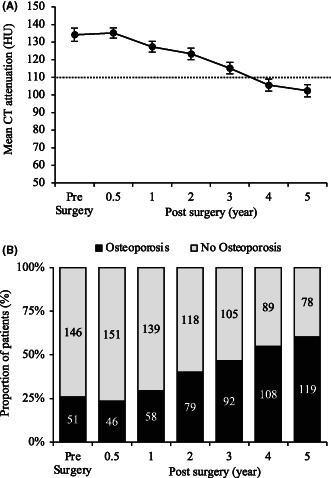

## 1 INTRODUCTION

Osteoporosis is a systemic skeletal disease characterized by low bone mass and microarchitectural deterioration in bone tissue. It is initiated by an imbalance between bone resorption and formation due to modifiable risk factors (inadequate nutritional absorption, weight loss, cigarette smoking, alcohol consumption, sarcopenia), nonmodifiable risk factors (older age, sex, white ethnic background, prior fracture), or secondary disease (corticosteroid use, hyperparathyroidism, vitamin D deficiency, diabetes).^1^, ^2^ In adults of all ages, bone mineral density (BMD) loss is associated with increased fracture risk, decreased bone strength, diminished quality of life, and increased mortality.^3^ Nguyen et al. demonstrated that a high rate of BMD loss was an independent predictor of all‐cause mortality in elderly men and women, independent of incident fractures and concomitant diseases.^4^

Patients with cancer also exhibit significant bone loss and fracture risks due to the disease, therapy for malignancy, and age‐related osteoporosis.^5^ Shapiro et al.^6^ showed that cancer therapy induced more rapid and severe bone loss than postmenopausal bone loss in women or that in normal age‐related osteoporosis in men. Moreover, cancer survivors often have additional risk factors for osteoporosis, such as advanced age, smoking, excessive alcohol consumption, impaired mobility, and reduced physical activity.^7^ Therefore, the risk of osteoporosis in patients with cancer should be carefully evaluated in order to provide appropriate treatment and ensure satisfactory quality of life in the long term.

Esophageal cancer is the eighth most common and the sixth most deadly cancer worldwide.^8^ Increased early detection and advances in multimodal treatments have improved survivorship among patients with esophageal cancer.^9^, ^10^ Moreover, the number of elderly patients with esophageal cancer undergoing radical surgery has increased with the increasing age of patients and technological innovations.^11^ Therefore, there is an increasing focus on qualitative outcomes and various issues impacting the long‐term quality of life in terms of survivorship.^12^, ^13^ Among them, osteoporosis has become an important concern for postoperative patients with esophageal cancer. However, few studies have investigated the association between esophageal cancer and osteoporosis. Moreover, there are no studies on the long‐term risk for osteoporosis and survival outcomes for patients with osteoporosis.

In the present study, we aimed to investigate long‐term BMD changes after esophagectomy and identify the risk factors for postoperative osteoporosis in patients with esophageal cancer. Furthermore, we investigated survival outcomes related to osteoporosis after radical esophagectomy.

## 2 PATIENTS AND METHODS

### 2.1 Patients

Between January 2010 and December 2016, 416 consecutive patients with thoracic esophageal cancer underwent radical esophagectomy at Osaka International Cancer Institute. Among those patients, 19 were lost to 5‐year follow‐up and 16 underwent non‐gastric tube reconstruction. After excluding these 35 patients, 381 patients were enrolled in a survival study. Of those patients, 143 patients died or had a recurrence within 2 years of surgery and 41 patients died or had a recurrence within 2–5 years of surgery. Finally, 197 patients who maintained a disease‐free status during the 5‐year follow‐up period were included in this retrospective study (Figure S1). Data on patient characteristics, surgical outcomes, clinicopathological features, and postoperative findings were reviewed from the medical reports. The patients were evaluated using esophagoscopy, computed tomography (CT), or positron emission tomography. The histopathological findings were classified according to the Union for International Cancer Control (UICC) Tumor, Nodes, Metastasis (TNM) classification system.^14^ Heavy drinking was defined as consumption of three units or more of alcohol per day; one unit equals 20 mg of pure alcohol. Postoperative complications were assessed by the Clavien–Dindo classification.^15^ Postoperative dysphagia was evaluated by a videofluoroscopic swallowing study as previously described.^16^ The rate of weight change was assessed relative to the preoperative weight. Since the median weight change at 1‐year after surgery was 88% in the present study, we defined severe weight loss as ≤85% weight change between the time of surgery and post‐surgery. Previous studies show that 10%–15% of unintentional weight loss is predictive of poorer clinical outcomes for patients with cancer and thus reduces the possibility of successful postoperative recovery.^17^

### 2.2 Preoperative treatment

In our institute, preoperative chemotherapy has been the standard treatment for advanced esophageal cancer, and it involves a triplet regimen with docetaxel (70 mg/m^2^), cisplatin (70 mg/m^2^), and 5‐fluorouracil (FU) (700 mg/m^2^/day). A doublet regimen with cisplatin (80 mg/m^2^) and 5‐FU (800 mg/m^2^/day) was selected depending on the patient's condition, age, and other comorbidities. Chemoradiation therapy (CRT) was selected as a preoperative treatment for patients with thoracic esophageal cancer with suspected cervical esophageal infiltration, disease refractory to initial chemotherapy, suspected cT4 disease, simultaneous double cancer, and for those who requested it. The CRT regimen comprised simultaneous radiation with cisplatin (70 mg/m^2^) and 5‐FU (700 mg/m^2^). External beam radiation therapy was administered at 1.8–2.0 Gy per fraction per day, five fractions per week, for a total dose of 40–60 Gy. The radiation field was designed to encompass the primary tumor and metastatic lymph nodes with an ample margin. The field of preventative irradiation included the bilateral supraclavicular fossae and superior mediastinal lymph nodes in patients with upper esophageal cancer, and the mediastinal lymph nodes in patients with middle or lower esophageal cancer. Surgery was scheduled within 6 weeks after completion of the last cycle of CRT or chemotherapy.

### 2.3 Radical esophagectomy

Standard procedures for treating thoracic esophageal cancer in this series of patients included transthoracic esophagectomy with upper, middle, and lower mediastinal lymphadenectomy; upper abdominal lymphadenectomy; reconstruction of the retrosternal and gastric tube; and anastomosis of the cervical incision. Cervical lymphadenectomy was not performed for patients with lower thoracic esophageal cancer when intraoperative histological examinations revealed negative findings for the recurrent laryngeal nerve lymph nodes. The operator chose to perform video‐assisted thoracic surgery according to the stage of cancer progression. Patients with a history of gastrectomy were reconstructed with the jejunum or ileocolic via an antethoracic route. The mediastinal route was selected only when the retrosternal route was difficult or impossible to use.

### 2.4 Bone mineral density (BMD)

BMD was assessed by CT attenuation values for the L1 vertebral body, as previously described by Pickhardt et al.^18^, ^19^ CT attenuation in a region of interest (ROI) was recorded in Hounsfield Units (HU), similar to the method used to generate BMD measurements using dual X‐ray absorptiometry (DXA), and it represents a linear transformation of the attenuation coefficient. A single oval click‐and‐drag ROI was placed over an area of trabecular bone in the vertebral body, and CT attenuation was measured between the T12 and L5 vertebral levels, with emphasis on the L1 measures. ROI placement near areas that would distort the BMD measurements (posterior venous plexus; focal heterogeneity or lesion, including compression fracture; and imaging‐related artifacts) was avoided. Osteoporosis was diagnosed using an L1 attenuation threshold of ≤110 HU, as previously reported.^18^, ^19^, ^20^ In the study by Pickhardt, these criteria specifically distinguished osteoporosis from osteopenia and normal BMD.^18^ L1 attenuation was measured before and after 6 months, and 1, 2, 3, 4, and 5 years after radical esophagectomy.

### 2.5 Statistical analysis

Results are expressed as median (range) for continuous variables or percentage for categorical variables. We retrospectively analyzed the associations between groups using χ^2^ tests, the Mann–Whitney *U* test, and one‐way repeated measures analysis of variance (anova). Cox proportional hazards regression analysis was used for the univariate and multivariate analyses. Disease‐free survival (DFS) was calculated from the date of surgery to the date of the first evidence of recurrence, death owing to any cause, or last follow‐up in patients without recurrence. Overall survival (OS) was calculated from the date of surgery to the time of death owing to any cause or to the last follow‐up. Cancer‐specific survival and non‐cancer‐specific survival were defined as the time from the date of diagnosis to the date of death due to esophageal cancer and diseases other than esophageal cancer, respectively. Survival analyses were performed using the Kaplan–Meier method, and survival curves were compared using log‐rank test. All statistical tests were two‐sided, and the threshold for statistical significance was set at *p* = 0.05. Statistical analyses were performed using JMP® Pro 15.1.0 (SAS Institute Inc.).

## 3 RESULTS

### 3.1 Clinicopathological characteristics and surgical outcomes

Table 1 shows the clinicopathological characteristics and surgical outcomes in 5‐year disease‐free patients. The median age was 65.0 (range 37–79) years. Risk factors for osteoporosis, such as female gender, heavy alcohol consumption, smoking history (current smoker/ex‐smoker), and respiratory dysfunction, were noted for 20% (*n* = 39), 43% (*n* = 84), 87% (*n* = 171), and 12% (*n* = 23) of patients, respectively. Retrosternal reconstruction was selected in most patients (81%, *n* = 160).

**TABLE 1:** Clinicopathological characteristics and surgical outcomes in 5‐year disease‐free patients

	*N* = 197
Patient characteristics at surgery	
Age, years, median (range)	65.0 (37–79)
≦54 *n* (%)	29 (15)
55–64z *n* (%)	64 (32)
65–74 *n* (%)	92 (47)
75≦ *n* (%)	12 (6)
Sex, *n* (%)	
Male/female	158/39 (80/20)
BMI, kg/m^2^, median (range)	21.7 (14.0–30.5)
Alcohol consumption, *n* (%)	
<3 units/3 units≦	113/84 (57/43)
Smoking history	
Current/ex/never	78/93/26 (40/47/13)
FEV1%, *n* (%)	
<70%/70%≦	23/174 (12/88)
Preoperative calcium, mg/dl, median (range)	
Preoperative phosphate, mg/dl, median (range)	
Preoperative treatment	
Non/CT/CRT, *n* (%)	104/77/16 (53/39/8)
Tumor location, *n* (%)	
Upper/middle/low	28/104/65 (14/53/33)
Histology, *n* (%)	
SCC/non‐SCC	187/10 (95/5)
cT, *n* (%)	
1/2/3/4	86/36/60/15 (44/18/30/8)
cN, *n* (%)	
0/1–3	118/79 (60/40)
cStage, *n* (%)	
I/II/III/IV	86/36/60/15 (44/18/30/8)
Surgery	
Surgical approach, *n* (%)	
Open/MIS	132/65 (67/33)
Lymphadenectomy, *n* (%)	
2‐Field/3‐Field	75/122 (38/62)
Reconstruction route, *n* (%)	
Antethoracic/retrosternal/mediastinal	8/160/29 (4/81/15)
Pathological characteristics	
pT, *n* (%)	
0/1/2/3	14/106/28/49 (7/54/14/25)
pN, *n* (%)	
0/1/2/3	110/68/16/3 (55/35/8/2)
pStage, *n* (%)	
0/I/II/III/IV	10/71/64/45/7 (5/36/32/23/4)
Postoperative outcomes	
Postoperative complication, *n* (%)	
Grade 0–2/grade 3≦	155/42 (79/21)
Postoperative dysphagia, *n* (%)	
Normal/mild/severe	123/66/8 (62/34/4)

Abbreviations: BMI, body mass index; FEV, forced expiratory volume; CT, chemotherapy; CRT, chemoradiation therapy; SCC, squamous cell carcinoma; MIS, minimally invasive esophagectomy.

### 3.2 Bone mineral density changes after esophagectomy

**FIGURE 1:**
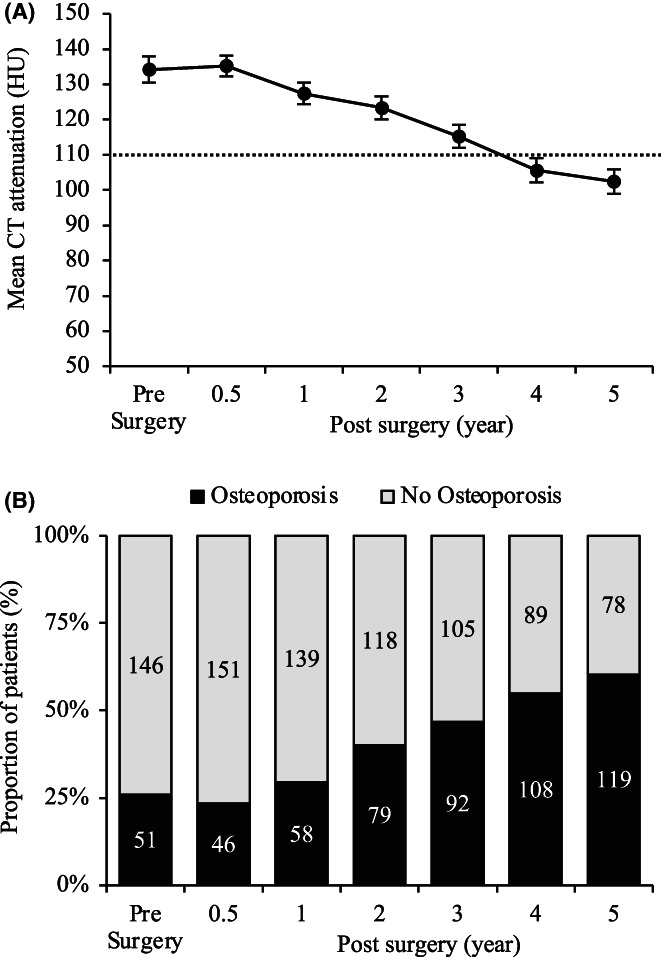
Changes in L1 attenuation on computed tomography (CT) over a period of 5 years after esophagectomy for thoracic esophageal cancer. (A) Changes in CT attenuation values for the L1 vertebral body after esophagectomy, (B) development of osteoporosis from before surgery to 5 years after surgery.

Figure 1 shows the changes in L1 attenuation values from before to 5 years after surgery. The median CT attenuation (HU) decreased annually after surgery, with values of 134.2 before surgery and 135.2, 127.4, 123.3, 115.2, 105.6, and 102.4 at 6 months, 1, 2, 3, 4, and 5 years after surgery, respectively (Figure 1A). Figure 1B shows the proportion of patients with osteoporosis diagnosed by an L1 attenuation value of ≤110 HU. The rate of osteoporosis increased annually from 1 year after esophagectomy. Fifty‐one patients (25.9%) were diagnosed with osteoporosis before surgery. After esophagectomy, the proportion of patients with osteoporosis gradually increased as follows: 6 months, 23.3% (*n* = 46); 1 year, 29.4% (*n* = 58); 2 years, 40.1% (*n* = 79); 3 years, 46.7% (*n* = 92); 4 years, 54.8% (*n* = 108); and 5 years, 60.4% (*n* = 119) (Figure 1B).

### 3.3 Body weight change after esophagectomy

**FIGURE 2:**
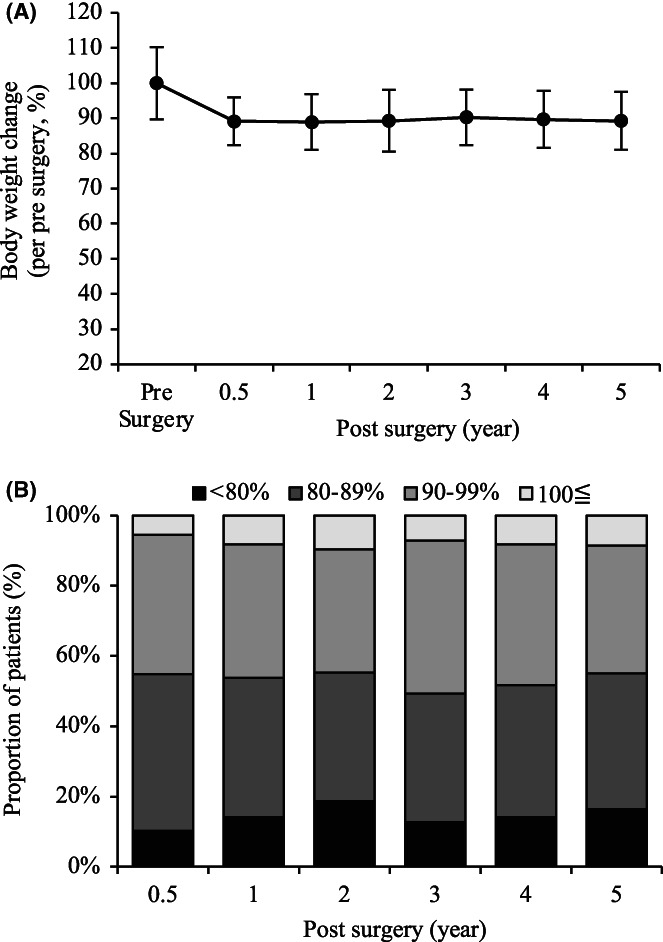
Evaluation of body weight over a period of 5 years after esophagectomy for thoracic esophageal cancer. (A) Body weight change after esophagectomy, (B) distribution of patients with postoperative body weight change.

We investigated body weight changes in patients with esophageal cancer. Body weight significantly decreased until 6 months after surgery, with no changes thereafter (Figure 2A). Body weight decreased in more than 90% of patients after esophagectomy; however, the proportion of patients with body weight change remained almost constant from 6 months after esophagectomy (Figure 2B).

### 3.4 Postoperative BMD changes corresponding to preoperative factors

**FIGURE 3:**
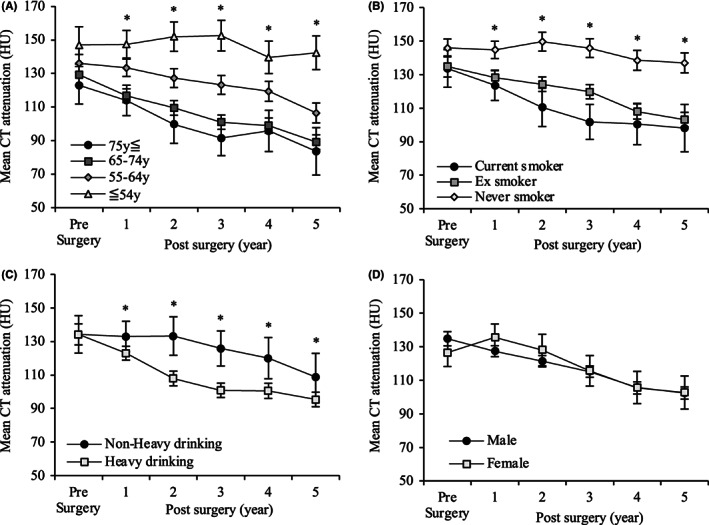
Bone mineral density changes over a period of 5 years after esophagectomy for thoracic esophageal cancer, according to different preoperative factors. (A) Age at surgery, (B) smoking history, (C) alcohol consumption, (D) sex (male/female) (*<0.050).

Figure 3 shows the postoperative BMD changes corresponding to preoperative factors. Postoperative BMD was not decreased in patients aged ≤54 years; however, it significantly decreased in each patient aged 65–74 and ≤75 years at 1–5 years after surgery compared with those aged ≤54 years, respectively. The median CT attenuation (HU) value at 5 years after surgery was 142.4, 106.5, 89.1, and 83.6 for patients aged ≤54, 55–64, 65–74, and ≥75 years, respectively (Figure 3A). Similarly, postoperative BMD was not decreased in those who had never smoked, whereas it significantly decreased in patients who were current smokers at 1–5 years after surgery and those who were ex‐smokers at 2–5 years after surgery compared with those who had never been smokers, respectively. The median CT attenuation (HU) value at 5 years after surgery was 137.0, 103.1, and 98.1 for never smokers, ex‐smokers, and current smokers at the time of surgery, respectively (Figure 3B). The postoperative BMD significantly decreased earlier in heavy drinkers compared with non‐heavy drinkers. The median CT attenuation (HU) values at 3 years after surgery was 100.9 and 125.8, respectively (Figure 3C). There were no significant differences in postoperative BMD changes according to the sex of the patient (Figure 3D).

### 3.5 Postoperative BMD changes corresponding to perioperative and postoperative factors

**FIGURE 4:**
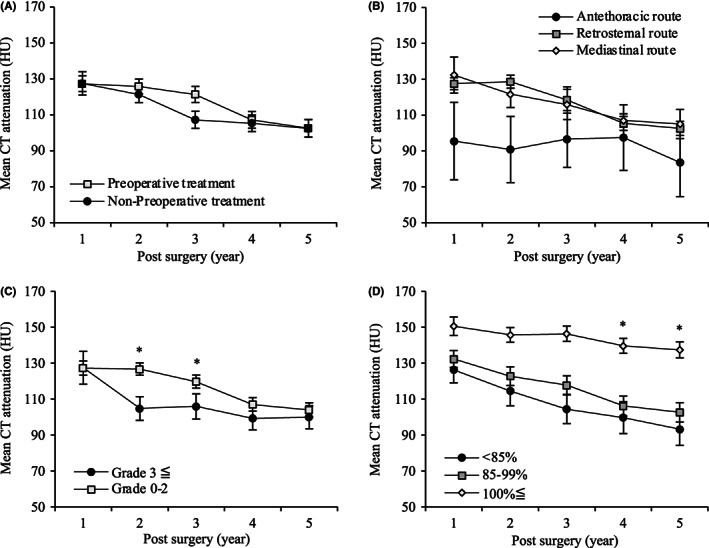
Bone mineral density changes over a period of 5 years after esophagectomy for thoracic esophageal cancer, according to different perioperative and postoperative factors. (A) Preoperative treatment, (B) reconstruction route, (C) postoperative complications (Clavien–Dindo classification), (D) body weight changes at 1 year after esophagectomy relative to the preoperative weight (*<0.050).

Figure 4 shows the postoperative BMD changes corresponding to each perioperative and postoperative factor. Postoperative BMD changes were not influenced by preoperative treatment and reconstruction route (Figure 4A,B). Patients with postoperative complications (CD3≤) showed significant BMD decrease at 2 and 3 years after esophagectomy compared with those with postoperative complications (CD0‐2). The median CT attenuation (HU) value at 2 and 3 years after surgery was 104.8 and 105.9 for patients with postoperative complications (CD3≤), respectively (Figure 4C). Postoperative BMD did not decrease rapidly in patients without weight loss at 1 year after surgery. It significantly decreased in each patient with weight change of 85%–99% and <85% at 4–5 years after surgery compared with those without weight loss, respectively, but the degree of weight loss did not affect postoperative BMD. The median CT attenuation (HU) value at 5 years after surgery was 137.4, 102.7, and 93.3 for patients with weight change of <85% (*n* = 59), 85–99% (*n* = 122), and 100% (*n* = 16), respectively (Figure 4D).

### 3.6 Evaluation of risk factors for osteoporosis at 5 years after esophagectomy

Potential risk factors for osteoporosis at 5 years after esophagectomy included preoperative characteristics, surgical factors, pathological characteristics, and postoperative outcomes. Univariate analysis showed that ages ≥65 at surgery, heavy drinking at diagnosis, smoking history (current smoker/ex‐smoker), percentage forced expiratory volume in the first second (FEV1%) <70% at diagnosis, and pT3 disease were significant risk factors for osteoporosis at 5 years after esophagectomy. Multivariate analysis revealed that age ≥65 years at surgery and smoking history were independent risk factors for osteoporosis at 5 years after esophagectomy (*p* < 0.001 and *p* = 0.024, respectively) (Table 2).

**TABLE 2:** Univariate and multivariate cox model analysis for osteoporosis 5 years after esophagectomy

	Univariate			Multivariate		
	Odds ratio	95% CI	*p*	Odds ratio	95% CI	*p*
Preoperative characteristics						
Age at surgery (≥65 years)	2.88	(1.59–5.20)	<0.001	2.96	(1.57–5.58)	<0.001
Sex (male)	1.23	(0.60–2.50)	0.569			
BMI (<17)	1.33	(0.32–5.47)	0.695			
Heavy alcohol consumption at diagnosis	2.08	(1.15–3.78)	0.016	2.13	(0.85–5.36)	0.107
Smoking history (current/Ex)	2.81	(1.20–6.58)	0.017	2.14	(1.10–4.14)	0.024
FEV1% at surgery (<70%)	3.52	(1.15–10.77)	0.028	2.67	(0.83–8.53)	0.098
Preoperative treatment	0.90	(0.51–1.60)	0.731			
Histology (SCC)	1.02	(0.28–3.73)	0.979			
Surgery						
Video‐assisted thoracic surgery	0.89	(0.48–1.62)	0.695			
Lymphadenectomy (2‐Field)	1.17	(0.65–2.10)	0.611			
Reconstruction route (antethoracic)	4.81	(0.58–39.90)	0.145			
Pathological characteristics						
pT 3	2.17	(1.06–4.42)	0.033	2.02	(0.94–4.32)	0.071
pN 1–3	1.13	(0.64–2.02)	0.671			
pStage III‐IV	1.50	(0.77–2.91)	0.237			
Postoperative outcomes						
Postoperative complication (Grade 3≦)	1.85	(0.88–3.89)	0.103			
Postoperative dysphagia	1.03	(0.57–1.85)	0.928			
>15% weight loss at 1 year after esophagectomy	1.41	(0.75–2.67)	0.286			

Abbreviations: BMI, body mass index; FEV, forced expiratory volume; SCC, squamous cell carcinoma.

### 3.7 Survival outcomes

Survival analysis was performed in 381 patients with 5‐year follow‐ups (Figure S1). Among them, 121 patients (32%) were diagnosed with osteoporosis before surgery. We compared preoperative patient characteristics between the osteoporosis and non‐osteoporosis groups in Table S1. Patients with preoperative osteoporosis were significantly older than those without osteoporosis (median 68.0 vs. 64.0, *p* < 0.001), while there were no significant differences in sex, BMI, preoperative biochemical micronutrients (calcium and phosphate), preoperative treatment, and pathological tumor‐node‐metastasis (pTNM) stage.

**FIGURE 5:**
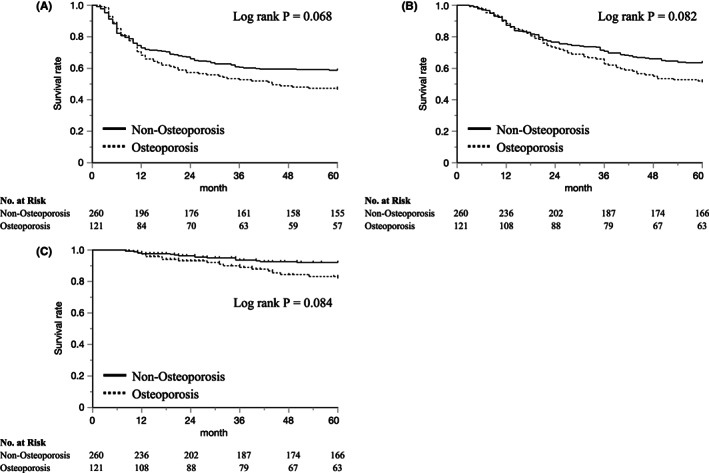
Survival analysis in patients with 5‐year follow‐up; (A) disease‐free survival (DFS), (B) overall survival (OS), (C) non‐cancer‐specific survival.

Patients with preoperative osteoporosis tended to have worse prognosis in DFS and OS than those without osteoporosis (log rank *p* = 0.068 and 0.082, respectively) (Figure 5A,B). The 5‐year OS was 51.2% in osteoporosis and 63.5% in non‐osteoporosis patients (Figure 5B). There was no significant difference in cancer‐specific OS between the preoperative osteoporosis and non‐osteoporosis groups (log rank *p* = 0.304) (Figure S2A). However, patients with preoperative osteoporosis tended to have worse prognosis in non‐cancer‐specific OS than those without osteoporosis (log rank *p* = 0.084) (Figure 5C).

## 4 DISCUSSION

In the present study, we found that postoperative BMD decreased annually over a period of 5 years after esophagectomy, and more than half of the patients developed osteoporosis by 5 years after esophagectomy. Multivariate analyses demonstrated that independent risk factors for osteoporosis at 5 years after esophagectomy were ages ≥65 years at surgery and smoking history. Moreover, patients with preoperative osteoporosis tended to have worse prognosis than those without osteoporosis and were more likely to die due to non‐esophageal cancer. This is the first study to report long‐term BMD changes after esophagectomy and survival outcomes related to osteoporosis in patients who undergo radical esophagectomy.

Habitual alcohol consumption and smoking are the most common risk factors for esophageal cancer.^21^ In addition, patients with esophageal cancer often develop various postoperative complications, such as dysphagia, pneumonia, micronutrient malnutrition, and weight loss.^16^, ^22^, ^23^ They may have an increased risk of developing osteoporosis after esophagectomy. However, BMD changes in postoperative patients with esophageal cancer have not been evaluated well. Postoperative osteoporosis in patients with esophageal cancer was previously investigated only by Elliott et al., who analyzed postoperative BMD changes in 65 consecutive disease‐free patients who underwent esophagectomy with gastric tube and had pathologically negative lymph node metastasis.^20^ They found that BMD declined at 1 and 2 years after surgery, relative to that before surgery [CT attenuation (HU): 144.3 ± 45.8 versus 128.6 ± 46.2 and 122.7 ± 43.5], with increased osteoporosis prevalence from 25% to 38% and 44%, respectively. However, the number of patients was limited, and the follow‐up period was short.^20^ In the present study, we included 197 consecutive patients who maintained a disease‐free status for 5 years after radical esophagectomy, regardless of histopathological findings. CT‐measured BMD showed no changes at 6 months after esophagectomy; from 1 year until 5 years after the surgery, it showed an annual decrease. The proportion of patients with osteoporosis was 25.9% at surgery and 60.4% at 5 years after surgery when the cutoff value for CT attenuation was set to ≤110 HU as previously reported.^18^, ^20^ These findings suggest that the long‐term incidence of osteoporosis after esophagectomy is high among esophageal cancer survivors.

Jang et al.^24^ established normative ranges for trabecular attenuation of the L1 vertebra across all adult ages as a quick reference to identify adults with low BMD who are at risk of developing osteoporosis using routine CT. Their study enrolled more than 20 000 adults and showed the median CT attenuation (HU) value for each age group using the same method used in the present study: 55–59 years, 163; 60–64 years, 153; 65–69 years, 139; 70–74 years, 132; 75–79 years, 117; and 80–84 years, 111. In the present study, preoperative BMD values were almost similar to the abovementioned reference values for adults in each age group; however, the decrease in BMD at 5 years after esophagectomy was significantly greater relative to the reference values for patients of all ages except those aged ≤54 years at the time of surgery. These results suggest that osteoporosis is more likely to develop in postoperative patients with esophageal cancer than in the general adult population.

Osteoporosis is generally initiated due to modifiable risk factors, nonmodifiable risk factors, or secondary disease.^1^ Among them, weight loss is considered one of the risk factors for the development of osteoporosis.^1^ In the present study, BMD significantly decreased in patients who lost weight at 1 year after the surgery. However, most patients with esophageal cancer experienced permanent weight loss after esophagectomy, and there was no association between the incidence of osteoporosis and the degree of weight loss. Moreover, BMD decreased year by year even though body weight remained almost unchanged from that at 6 months after esophagectomy. Therefore, the incidence of postoperative osteoporosis might be associated with other factors in addition to weight loss. The present study demonstrated that age, heavy alcohol consumption, smoking history, and pT stage could be associated with a BMD decrease after esophagectomy. Since most patients with esophageal cancer have habitual alcohol consumption and smoking histories before surgery, these factors may be more closely involved in causing further reduction in BMD after esophagectomy. Moreover, patients with esophagectomy may have problems, such as nutrient malabsorption related to bone formation, sarcopenia, and other diseases, in addition to poor nutritional intake. Therefore, future studies on postoperative patient factors might be important when considering future measures to prevent osteoporosis after esophagectomy.

Bone mineral density loss is associated with increased mortality in addition to increased fracture risk, decreased bone strength, and diminished quality of life.^3^, ^4^ Nabeya et al. showed that ICTP, a marker of bone resorption, was a prognosticator in patients after esophagectomy.^25^ However, there are no studies on survival outcomes related to osteoporosis in postoperative patients with esophageal cancer. In the present study, preoperative osteoporosis patients had worse prognoses in DFS, OS, and non‐cancer specific OS than non‐osteoporosis ones. Although there were no differences in the survival curve within 1 year after surgery, the survival rate for osteoporotic patients declined year by year. Moreover, the results of non‐cancer specific OS suggest that patients with preoperative osteoporosis have a higher risk of death from other diseases. Therefore, long‐term diminished quality of life due to BMD loss may be associated with prognosis in postoperative patients with esophageal cancer. We also investigated survival outcomes related to postoperative osteoporosis in 2‐year disease‐free patients. However, no significant differences in OS were observed in 238 patients (Figure S2B) because of the shorter observation period compared to survival analysis in 5‐year disease‐free patients.

Patients with gastrectomy often have bone metabolism disorders. The prevalence rate of osteoporosis in gastric cancer survivors who undergo gastrectomy is reported to be approximately 38%–55%^26^; this rate is comparable with the prevalence of osteoporosis in esophageal cancer survivors after esophagectomy in the present study. Most patients with gastric cancer experience rapid weight loss (from 5% to 15%) during the immediate postoperative period, similar to postoperative patients with esophageal cancer. Changes in the absorption of the nutrients such as calcium and vitamin D have been implicated in osteoporosis after gastric cancer surgery.^26^ Moreover, weight loss in patients with gastrectomy can, directly or indirectly, impact bone tissue by increasing bone resorption due to reduced mechanical loading or causing dysregulation of hormonal pathways (e.g. those of ghrelin, leptin, and adiponectin).^27^, ^28^, ^29^ On the other hand, the absorption of vitamin D and calcium in postoperative patients with esophageal cancer remains unclear. In the present study, no differences in preoperative calcium and phosphate were observed between patients with preoperative osteoporosis and those without osteoporosis (Table S1). However, postoperative changes could not be investigated due to the lack of data on postoperative calcium and phosphate levels. Further studies should evaluate postoperative biochemical micronutrients to determine the association between bone remodeling, micronutrient deficiency, or bone metabolism changes and esophagectomy in cases of esophageal cancer.

This study has several limitations. First, it is a retrospective study conducted at a single institution. However, no study has evaluated the long‐term risk of osteoporosis after esophagectomy. Also, the present study included a significantly larger number of patients with esophageal cancer who underwent radical esophagectomy than the previous study by Eliott et al.^20^ Second, central DXA of the hips and lumbar spine is recognized as the reference standard for diagnosing osteoporosis worldwide.^30^ The present study assessed BMD using L1 CT attenuation values. Pickhardt et al. demonstrated that trabecular attenuation values for the vertebral bodies on CT scans could be used to estimate BMD.^16^, ^17^ Because normative ranges for L1 trabecular attenuation have been established for all ages, direct HU measurements from diagnostic CT scans can be helpful and reliable for opportunistic use in osteoporosis screening.^24^ Third, although we revealed the incidence of osteoporosis after esophagectomy, we could not investigate bone fracture and the long‐term quality of life in patients with osteoporosis after esophagectomy. In general, bone loss enhances bone fragility and increases fracture risk, which results in impaired quality of life.^3^ Moreover, osteoporosis can be a predictor of all‐cause mortality in elderly men and women.^3^, ^4^ Further studies are required to clarify the association between osteoporosis and quality of life and investigate the effect of interventional treatment for osteoporosis in postoperative patients with esophageal cancer.

In conclusion, the present study demonstrated that postoperative BMD assessed by CT showed an annual decrease after esophagectomy, with the rate of patients with osteoporosis increasing from 25.9% before surgery to 60.4% at 5 years after surgery. BMD decrease after esophagectomy might be associated with preoperative patient factors and postoperative factors. Moreover, patients with preoperative osteoporosis had possibly worse prognosis than those without osteoporosis. These results provide evidence that can aid in personalizing the long‐term follow‐up of esophageal cancer survivors who undergo esophagectomy. Furthermore, adequate surveillance and appropriate interventional treatments are required in patients with a high risk of developing osteoporosis, such as the elderly or those with a smoking history.

## DISCLOSURE

Conflict of Interest: The authors declare no conflict of interests for this article.

Ethical Statements: The protocol for this research project has been approved by a suitably constituted Ethics Committee of the institution (Human Ethics Review Committee of Osaka International Cancer Institute, Approval No. 18033–4), and it conforms to the provisions of the Declaration of Helsinki (If cases are involved).

## Supporting information


Table S1

Figure S1

Figure S2
