# Supplementary material for: Long‐term changes in bone mineral density in postoperative patients with esophageal cancer
Source: Ann Gastroenterol Surg. 2022 Nov 29;7(3):419–29. doi: 10.1002/ags3.12640 (PMC10154838; doi:10.1002/ags3.12640)
Supplement: Supplementary file 1 — Table S1 [file AGS3-7-419-s002.docx]

Supplemental Table 1. Comparison of preoperative patient characteristics in patients with 5-year follow-up

|  | Osteoporosis N=121 | | Non-Osteoporosis N=260 | | P |
| --- | --- | --- | --- | --- | --- |
| Age, years, median (range) | 68.0 | (49 – 85) | 64.0 | (37 – 80) | <0.001 |
| Sex, n, (%) |  |  |  |  | 0.435 |
| Male / Female | 95 / 26 | (79 / 21) | 213 / 47 | (82 / 18) |  |
| BMI, kg/m^2^, median (range) | 22.0 | (16.5 – 27.7) | 21.6 | (14.0 – 30.5) | 0.730 |
| Preoperative calcium, mg/dL, median (range) | 8.8 | (7.2 – 10.1) | 8.9 | (6.7 – 10.4) | 0.811 |
| Preoperative phosphate, mg/dL, median (range) | 3.6 | (2.0 – 4.8) | 3.5 | (2.0 – 4.5) | 0.669 |
| Preoperative treatment, n (%) | 68 / 53 | (56 / 44) | 143 / 117 | (55 / 45) | 0.827 |
| Tumor location, n (%) |  |  |  |  | 0.463 |
| Upper / Middle / Low | 19 / 64 / 38 | (16 / 53 / 31) | 46 / 124 / 90 | (18 / 48 / 34) |  |
| Histology, n (%) |  |  |  |  | 0.502 |
| SCC / non-SCC | 116 / 5 | (96 / 4) | 245 / 15 | (95 / 5) |  |
| pT |  |  |  |  | 0.933 |
| 0 / 1 / 2 / 3 / 4 | 9 / 52 / 16 / 41 / 3 | (7 / 43 / 13 / 34 / 3) | 17 / 105 / 33 / 100 / 5 | (7 / 40 / 13 / 38 / 2) |  |
| pN |  |  |  |  | 0.589 |
| 0 / 1 / 2 / 3 | 58 / 37 / 17 / 9 | (48 / 31 / 14 / 7) | 105 / 92 / 41 / 22 | (40 / 35 / 16 / 9) |  |
| pStage |  |  |  |  | 0.561 |
| 0 / I / II / III / IV | 6 / 33 / 35 / 29 / 18 | (5 / 27 / 29 / 24 / 15) | 9 / 63 / 75 / 82 / 31 | (3 / 24 / 29 / 32 / 12) |  |
| BMI, body mass index; SCC, squamous cell carcinoma | | | | | |
