# Supplementary figures and images for: Long‐term changes in bone mineral density in postoperative patients with esophageal cancer
Source: Ann Gastroenterol Surg. 2022 Nov 29;7(3):419–29. doi: 10.1002/ags3.12640 (PMC10154838; doi:10.1002/ags3.12640)

Supplemental Figure 1

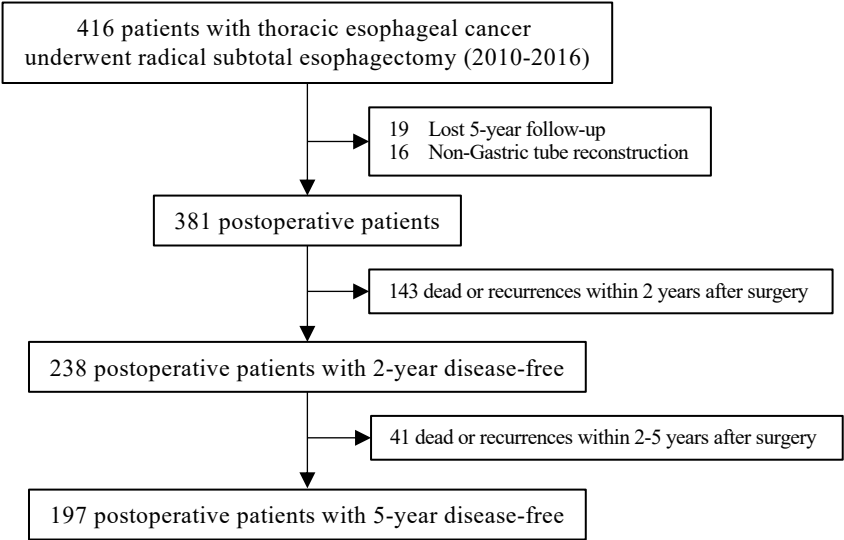

Supplement: Supplementary file 2 — Figure S1 [file AGS3-7-419-s003.pdf]

Supplemental Figure 2.

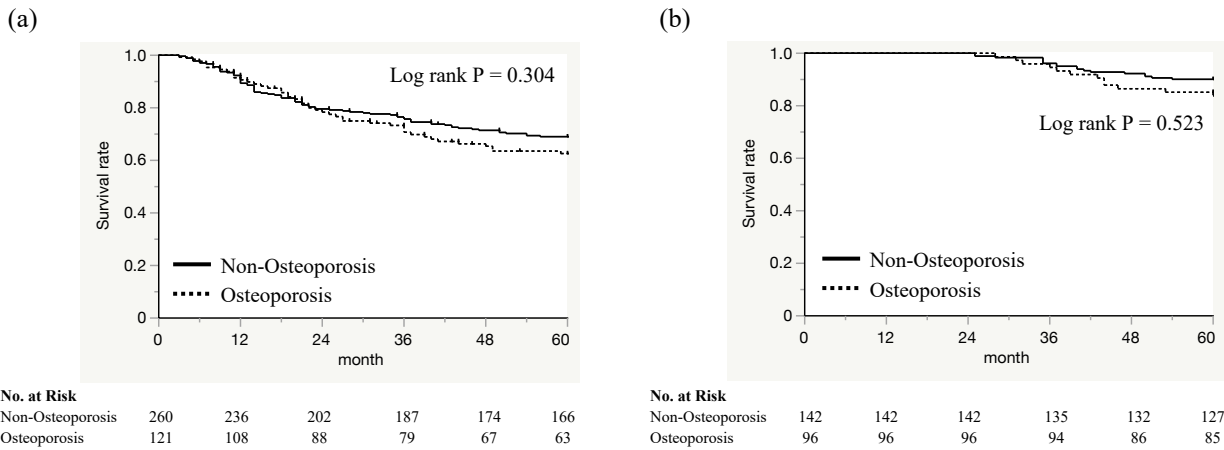

Supplement: Supplementary file 3 — Figure S2 [file AGS3-7-419-s001.pdf]
